# 

**Published:** 1867-05

**Authors:** 


### CONTENTS.

### ORIGINAL COMMUNICATIONS.

PAGE.

ARTICLE I.—Hypodermic Administration of Medical Agents. By WM. A. GREENE, M. D., of Americus, Ga ..... 97

ARTICLE II.—Reduction of an unusual Dislocation of both Bones of the Fore Arm at the Elbow Joint, complicated with the fracture of the internal condyle of the humerus and of the tip of the Caronoid process of the Ulna, two months after the receipt of the injury. Reported by A. W. GRIGGS, M. D., of West-Point, Ga ..... 108

### SELECTIONS.

A case of Progressive Muscular Atrophy from Lead-Poisoning ..... 112
A case of Hysterical Paraplegia ..... 114
Complete Transverse Division of the Urethra by a kick of a horse on the Perineum, &c ..... 116
Treatment of Acute Orchitis by the alternate application of Heat and Cold ..... 118
A case of Vaginismus,—Treatment of, &c ..... 120
On the Hereditary Transmission of Pulmonary Tuberculosis ..... 121

### EDITORIAL AND MISCELLANEOUS.

The Study of Practical Anatomy during the summer months ..... 130
Medical Association of Georgia ..... 131-132
Dr. Storer's Lectures ..... 144

